# The yeast AMP-activated protein kinase Snf1 phosphorylates the inositol polyphosphate kinase Kcs1

**DOI:** 10.1016/j.jbc.2024.105657

**Published:** 2024-01-13

**Authors:** Sham Sunder, Joshua S. Bauman, Stuart J. Decker, Alexandra R. Lifton, Anuj Kumar

**Affiliations:** Department of Molecular, Cellular, and Developmental Biology, University of Michigan, Ann Arbor, Michigan, USA

**Keywords:** yeast, *Saccharomyces cerevisiae*, AMPK, pseudohyphal growth, filamentous growth, Snf1, inositol polyphosphate

## Abstract

The yeast Snf1/AMP-activated kinase (AMPK) maintains energy homeostasis, controlling metabolic processes and glucose derepression in response to nutrient levels and environmental cues. Under conditions of nitrogen or glucose limitation, Snf1 regulates pseudohyphal growth, a morphological transition characterized by the formation of extended multicellular filaments. During pseudohyphal growth, Snf1 is required for wild-type levels of inositol polyphosphate (InsP), soluble phosphorylated species of the six-carbon cyclitol inositol that function as conserved metabolic second messengers. InsP levels are established through the activity of a family of inositol kinases, including the yeast inositol polyphosphate kinase Kcs1, which principally generates pyrophosphorylated InsP_7_. Here, we report that Snf1 regulates Kcs1, affecting Kcs1 phosphorylation and inositol kinase activity. A *snf1* kinase-defective mutant exhibits decreased Kcs1 phosphorylation, and Kcs1 is phosphorylated *in vivo* at Ser residues 537 and 646 during pseudohyphal growth. By *in vitro* analysis, Snf1 directly phosphorylates Kcs1, predominantly at amino acids 537 and 646. A yeast strain carrying *kcs1* encoding Ser-to-Ala point mutations at these residues (*kcs1*-S537A,S646A) shows elevated levels of pyrophosphorylated InsP_7_, comparable to InsP_7_ levels observed upon deletion of *SNF1*. The *kcs1*-S537A,S646A mutant exhibits decreased pseudohyphal growth, invasive growth, and cell elongation. Transcriptional profiling indicates extensive perturbation of metabolic pathways in *kcs1*-S537A,S646A. Growth of *kcs1*-S537A,S646A is affected on medium containing sucrose and antimycin A, consistent with decreased Snf1p signaling. This work identifies Snf1 phosphorylation of Kcs1, collectively highlighting the interconnectedness of AMPK activity and InsP signaling in coordinating nutrient availability, energy homoeostasis, and cell growth.

AMP-activated kinase (AMPK) is a central regulator of energetics (1, 2, 3, 4). In response to increased levels of AMP/ADP, indicative of a decreased cellular energy state, AMPK promotes ATP production through a regulatory program activating catabolism and inhibiting anabolism (5, 6, 7). AMPK is highly conserved in eukaryotes, and the yeast protein, Snf1, was the first kinase of the subsequently termed AMPK family to be identified (8, 9). Although Snf1 is not allosterically activated by AMP, its activity is correlated with high ratios of AMP to ATP (9). Snf1 is activated under conditions of glucose limitation and in response to external stressors, including salt stress, heat shock, alkalinity, and oxidative stress (10, 11, 12, 13). Snf1 is required for the wild-type response to glucose limitation and for the utilization of alternate carbon sources (14, 15, 16, 17). The *SNF1* gene derives its name (*S*ucrose *N*on*F*ermenting) from its loss-of-function phenotype, with *snf1* mutants able to metabolize glucose but unable to grow anaerobically on sucrose (18). Snf1 functions as the catalytic α-subunit of a heterotrimeric complex, with one of three β-subunits (Sip1p, Sip2p, or Gal83p), and the γ-subunit Snf4p (19, 20, 21). Under conditions of high glucose, Snf1 is negatively regulated through several mechanisms, including autoinhibition by internal binding of its amino and carboxy terminal regions (22, 23, 24, 25). In low glucose, Snf4 binds and relieves Snf1 autoinhibition (23). The kinases Sak1, Elm1, and Tos3 can each phosphorylate Snf1 at Thr 210, activating Snf1 and a complex downstream signaling network encompassing proteins that contribute to the metabolism of alternative carbon sources and fatty acids, gluconeogenesis, carbohydrate storage, and respiration (23, 26, 27, 28). The activated Snf1 complex functions in glucose derepression through activation of the transcription factors Cat8 and Sip4 and inhibition of the repressor Mig1 (1, 29, 30, 31).

The Snf1 signaling network is central in the stress-responsive transition of yeast cells from a planktonic growth mode to one characterized by the formation of extended and invasive multicellular pseudohyphal filaments (32, 33). Yeast pseudohyphal growth is induced under conditions of nitrogen or glucose limitation, with yeast cells undergoing changes in morphology, polarity, and adhesion to form connected and elongated chain-like filaments (34, 35, 36, 37). These pseudohyphal filaments, so named because they superficially resemble hyphal tubes evident in filamentous fungi, are presumed to represent a foraging mechanism by which nonmotile yeast can scavenge a broader surface area for nutrients (34). Pseudohyphal growth in *Saccharomyces cerevisiae* informatively models related processes of filamentous development required for virulence in the opportunistic human pathogen *Candida albicans* (38, 39, 40, 41). Yeast pseudohyphal growth is regulated through a complex signaling network encompassing the Kss1 mitogen-activated protein kinase (MAPK) pathway, the Ras2/cAMP-dependent protein kinase A (PKA) pathway, the target of rapamycin (TOR) pathway, and the aforementioned AMPK/Snf1 pathway (42, 43, 44, 45, 46, 47). In filamentous strains of *S. cerevisiae*, disruption or deletion of *SNF1* results in decreased surface-spread pseudohyphal growth and reduced invasive filamentation. Snf1-Gal83 activates transcription of the pseudohyphal growth cell surface adhesin Flo11 through inhibitory phosphorylation of the negative regulators Nrg1 and Nrg2 (32, 48). Snf1 is required for the wild-type phosphorylation of a large set of proteins (49, 50, 51), although we have not yet determined the full scope of Snf1 signaling in pseudohyphal growth.

The genetic basis of yeast pseudohyphal growth is expansive (37, 52, 53, 54), and genes encoding components of the inositol polyphosphate (InsP) family of metabolic second messengers are required for wild-type pseudohyphal filamentation (55). InsP signaling mediates numerous cell processes of growth and differentiation (56, 57, 58, 59). InsPs are a family of highly charged lipid-derived metabolites, constituting phosphorylated forms of the six-carbon cyclitol *myo*-inositol (Fig. 1*A*) (60, 61, 62, 63, 64). Inositol is processed to the glycerophospholipid phosphatidylinositol (PI) and subsequently to phosphoinositides, such as PI 4,5-bisphosphate (PIP_2_). Stimulated PIP_2_ is cleaved by Phospholipase C to yield the soluble second messenger inositol 1,4,5-trisphosphate (InsP_3_) (65). In many eukaryotes, InsP_3_ interacts with a channel receptor to release intracellular calcium stores; however, InsP_3_-mediated calcium release has not been observed in yeast (66). Despite this, the yeast biosynthetic InsP pathway is very similar in composition to orthologous eukaryotic InsP pathways (61, 64, 67). InsP_3_ is phosphorylated sequentially by inositol kinases to yield a series of inositol polyphosphate species. In *S. cerevisiae*, the inositol kinase Arg82 sequentially phosphorylates InsP_3_ at the three- and 5-carbon positions of inositol, producing inositol InsP_5_ (61, 68, 69). The inositol 2-kinase Ipk1 converts InsP_5_ to InsP_6_ (70). Although much less abundant than InsP_6_ and subject to high turnover, the doubly charged inositol pyrophosphates have emerged as important signaling molecules required for the wild-type regulation of phosphate homeostasis in eukaryotes as well as cellular responses to osmotic, heat, and oxidative stress (58, 71, 72, 73). The InsP_6_ kinases Kcs1 and Vip1 catalyze formation of InsP_7_ isomers and InsP_8_ (61, 64). Kcs1 also produces pyrophosphorylated InsP species from InsP_5_, generating 5PP-InsP_4_, which, in turn, can be phosphorylated by Vip1 to yield (PP)_2_-InsP_3_ (62). The physiological significance of these latter pyrophosphorylated species has not been conclusively determined. Notably, deletion of any gene encoding an inositol kinase perturbs pseudohyphal growth (55).

**Figure 1:**
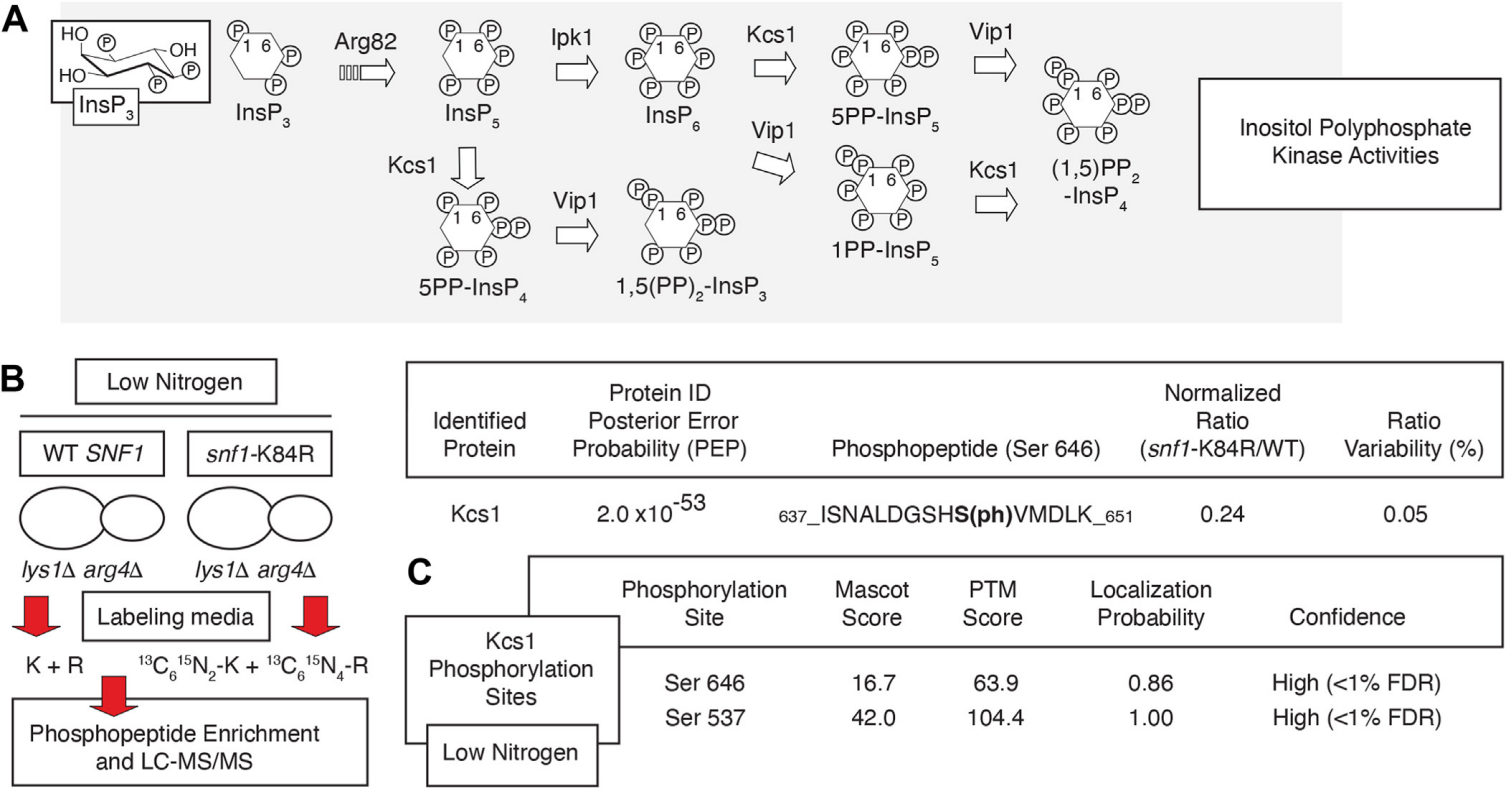
**Kcs1 is a phosphoprotein that undergoes Snf1-dependent phosphorylation *in vivo*.***A*, diagram of the inositol polyphosphate biosynthetic pathway. The boat conformation for InsP_3_ is shown below a flat representation of InsP_3_ with the one and six positions of inositol labeled. Proteins catalyzing each reaction are indicated. *B*, mass spectrometry identifies decreased phosphorylation of Kcs1 Ser 646 under steady-state conditions of nitrogen limitation in a strain with a kinase-defective allele of *snf1*. For quantitative mass spectrometry by SILAC, both strains for analysis contained deletions of *lys1* and *arg4* and were grown in media with either normal Lys and Arg or in media with the indicated stable isotopic forms. The wild-type strain is deleted for *snf1* and carries a low-copy centromeric plasmid containing the wild-type *SNF1* sequence with native promoter. The plasmid in the test strain encodes a kinase-defective allele of *snf1* with the indicated substitution of Arg for Lys 84. The posterior error probability (PEP) is the probability that the observed peptide-spectrum match for identification of the protein is incorrect. The ratio of phosphorylated peptide in the mutant and wild-type strains, normalized against total protein, is indicated. *C*, mass spectrometry of a wild-type strain of the filamentous Σ1278b background grown under conditions of nitrogen limitation identified phosphorylation of Kcs1 at Ser residues 537 and 646. The probability of correct amino acid identification is indicated, along with confidence and false discovery rate.

Several lines of evidence indicate that *KCS1* and inositol pyrophosphate levels contribute to the AMPK-regulated yeast pseudohyphal growth response. Under conditions of nitrogen limitation, deletion of *KCS1* results in decreased pseudohyphal growth relative to wild type (55). Overexpression of *KCS1* is sufficient to allow pseudohyphal filamentation under otherwise repressive conditions with normal levels of the nitrogen source ammonium sulfate (55). As reported by Bang *et al.* (74), inositol polyphosphate multikinase (IMPK), orthologous to Arg82 in yeast, physiologically binds AMPK, with binding enhanced by glucose availability and phosphorylation of IMPK at Tyr 174. Collectively, the data suggest interconnections between AMPK signaling, pseudohyphal growth, and inositol polyphosphate signaling but do not define a mechanism through which AMPK regulates InsP signaling. Here, we investigate this regulatory relationship through data that identify Kcs1 as a substrate of Snf1p and indicate the phenotypic effects of Snf1p-dependent Kcs1 phosphorylation.

## Results

### Snf1 regulation of Kcs1 phosphorylation

Under conditions of nitrogen limitation that induce pseudohyphal growth, Snf1 kinase activity is required for wild-type phosphorylation of Kcs1. By SILAC-based mass spectrometry of wild-type yeast compared against a strain with a kinase-defective allele of Snf1 (*snf1-*K84R), we identified hypophosphorylation of Kcs1 at Ser 646 (Fig. 1*B*) (51, 75). We applied mass spectrometric analysis of a filamentous wild-type yeast strain grown under conditions of nitrogen limitation to identify phosphorylation sites during pseudohyphal growth, and we identified phosphorylation of Kcs1 at residues Ser 537 and Ser 646 (Fig. S1*C* and File S1). Both phosphorylated residues are found outside of the putative Kcs1 glycine-rich G-loop (roughly at AA 337), important for ATP binding and phosphate transfer in protein kinases, and the Kcs1 kinase domain (AA 759–956), as identified in the InterPro/Pfam database (Fig. S1*A*) (76). Kcs1 is a known phosphoprotein that undergoes extensive phosphorylation, and the Kcs1 Ser 537 and 646 residues have been identified independently as phosphorylation sites in several studies (Fig. S1*B*) (51, 77, 78, 79, 80). The consensus sequence motif for AMPK substrates is shown in Fig. S1*C* (81, 82, 83).

**Figure 2:**
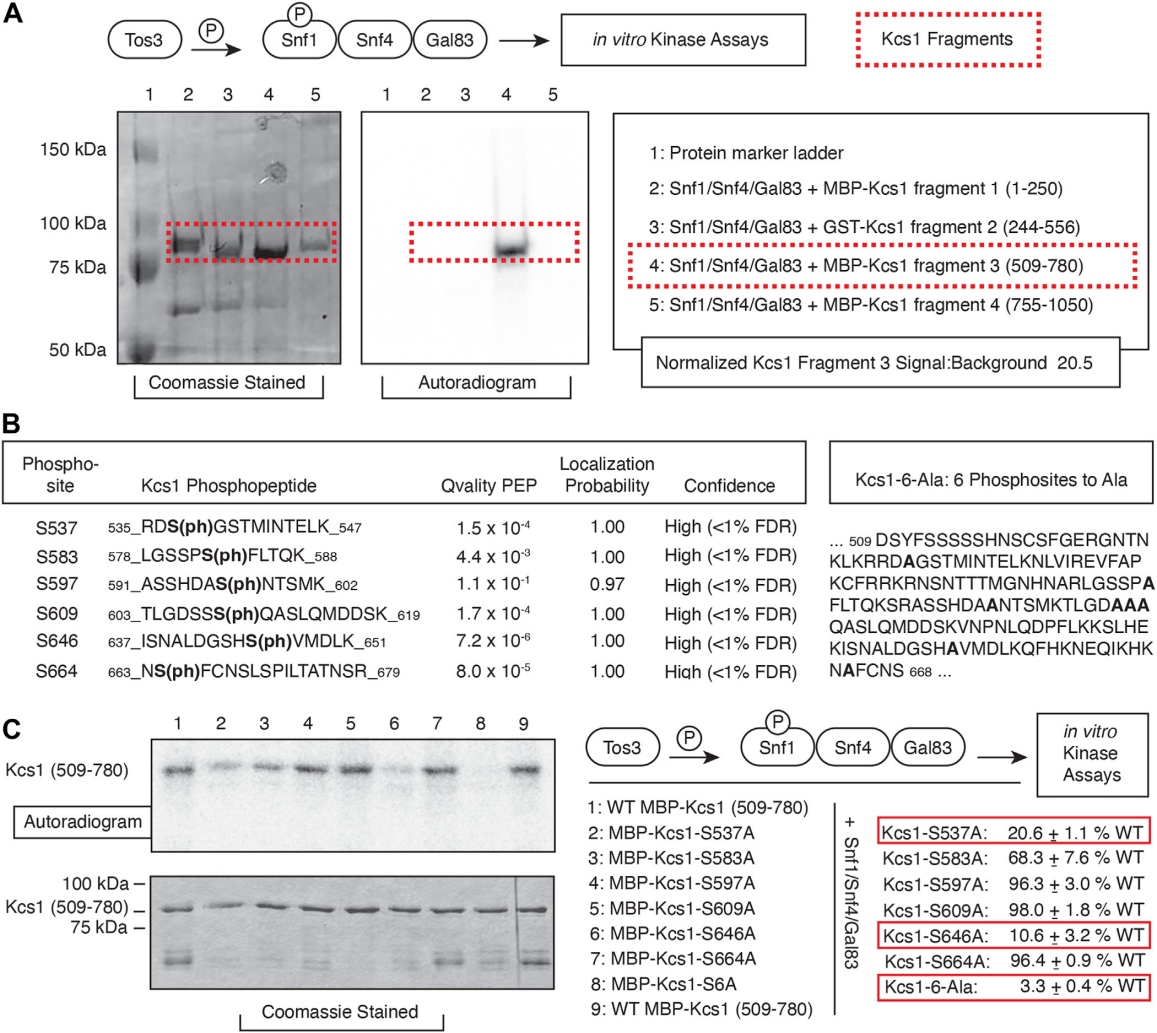
**Kcs1 is directly phosphorylated by purified Snf1 complex *in vitro*.***A*, proteins expressed for purification of active Snf1 kinase complex are diagrammed to the left. *In vitro* kinase assays are shown to identify Snf1 kinase phosphorylation of an MBP-Kcs1 chimera encoding Kcs1 amino acids 509 to 780. Bands representing phosphorylated MBP-Kcs1 fusions are boxed, as are bands corresponding to a GST fusion encoding Mig1 amino acids 202 to 414. Mig1 is a known substrate of Snf1. Kcs1 signal was quantified against the background signal over an identical area in the respective lane. *B*, Kcs1 sites phosphorylated by purified Snf1 kinase complex *in vitro* are shown in bold with flanking sequence. The probability of correct phosphosite determination and confidence indication with a false discovery rate is listed. The posterior error probability determined by the Qvality C++ program is shown. Six sites of phosphorylation by Snf1 were identified, and we mutated each of the sites individually to encode alanine. The *kcs1*-6-Ala mutant encodes a protein fragment with all six of the sites modified to encode alanine. *C*, Coomassie-stained gel and corresponding autoradiogram indicating *in vitro* kinase assay results for Snf1 phosphorylation of the wild-type Kcs1 fragment and site-directed phosphorylation site mutants. Densitometry was used to quantify radiolabeled band intensity relative to protein levels. Mean values relative to wild-type and standard deviation from three replicates of the assays are shown. Mutation of Ser 537 and Ser 646 to Ala strongly reduced Kcs1 fragment phosphorylation, as did mutation of all the sites to Ala.

We assessed the likelihood that Snf1 directly phosphorylates Kcs1 by purifying Snf1 and Kcs1 for *in vitro* analysis of Snf1 kinase activity. We generated active Snf1 in bacteria through co-expression with its upstream kinase Tos3, the Snf1 complex β-subunit Gal83, and the γ-subunit Snf4 (Fig. 2*A*). For this purpose, we used a two-plasmid system allowing for the heterologous expression of these yeast genes in *E. coli* (84). We purified a complex of His-tagged Snf1 with Snf4 and Gal83 by passage over a nickel column, yielding active Snf1 heterotrimer. We cloned the *KCS1* coding sequence in four fragments into bacterial expression vectors for subsequent purification as MBP- or GST-fusions (Fig. 2*A*). We expressed and purified a GST-Mig1 chimera encoding amino acids 202 to 414 of Mig1. Mig1 is a known substrate of Snf1, and this construct serves as a positive control. We also cloned and purified Plc1 as two GST-fusion fragments for analysis of Snf1 phosphorylation (Fig. S2) to consider the possibility that Snf1 may target Plc1 as an additional control point in modulating InsP signaling. Of these proteins, we detected phosphorylation of GST-Mig1 and the third fragment of Kcs1 (MBP-Kcs1 encoding AA 509–780) by the Snf1 kinase complex (Fig. 2, *A*). We mapped phosphorylation sites on the third Kcs1 fragment by mass spectrometry, and the sequence context of the six identified phosphorylated residues (Ser 537, Ser 583, Ser 597, Ser 609, Ser 646, and Ser 664) is indicated in Figure 2*B*.

We determined the degree to which these sites contributed to *in vitro* phosphorylation of Kcs1p by individually mutating each of the six sites to non-phosphorylatable alanine. In complement, we generated an expression construct with all six phosphorylation sites modified to encode alanine (*kcs1-*6-Ala). Ser 609 is the third of three consecutive serine residues in Kcs1 (AA 607–609). Although mass spectrometric analysis identified Ser 609 as the only phosphorylated residue among the three, we mutated all three serine residues to alanine in *kcs1-*6-Ala. Phosphorylation by the Snf1 complex was assayed as before. Kcs1 phosphorylation was most strongly affected by the substitution of alanine for Ser 537 and Ser 646, with phosphorylation decreased to approximately 21% and 11% of wild-type levels, respectively (Fig. 2*C*). Alanine substitution at all identified phosphorylation sites in *kcs1-*6-Ala eliminated roughly 97% of Kcs1 phosphorylation by Snf1 (Fig. 2*C*). In total, the *in vitro* and *in vivo* data are consistent with Kcs1 being a substrate of Snf1 and identify principal Snf1-dependent phosphorylation at Kcs1 Ser 537 and Ser 646.

### Kcs1 Ser 537 and Ser 646 are required for wild-type levels of inositol polyphosphates

**Figure 3:**
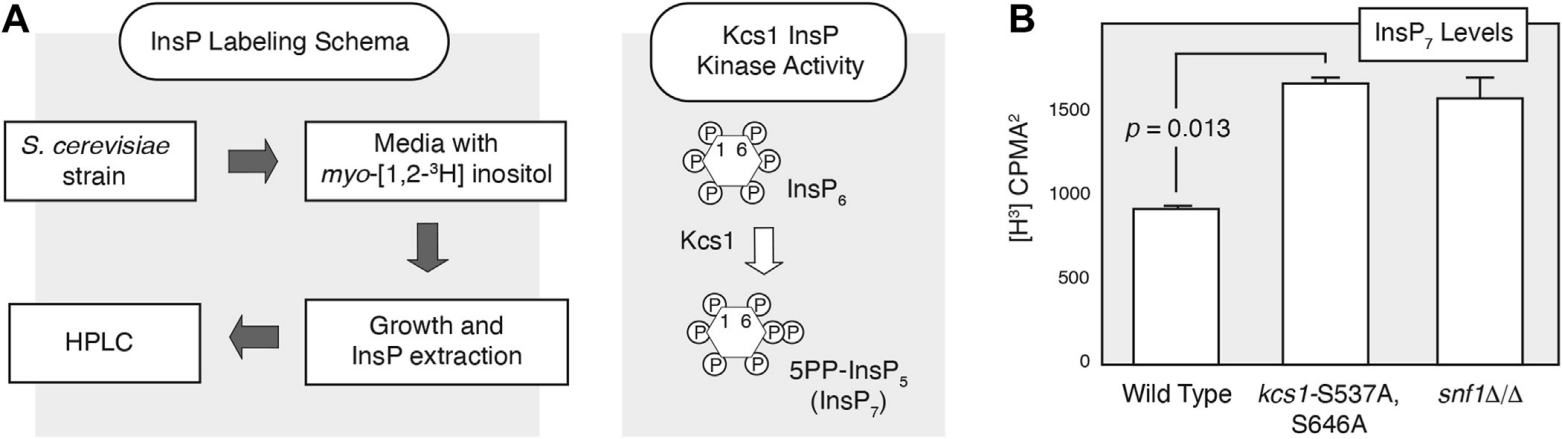
**InsP**_**7**_**levels are elevated relative to wild type in the *kcs1*-S537A,S646A mutant.***A*, schema for radiolabeling of inositol polyphosphates by incorporation of *myo*[1,2-H^3^]-inositol and diagram highlighting the predominant activity of Kcs1 in generating the 5PP-InsP_5_ isoform of InsP_7_. *B*, InsP levels were measured in a yeast strain with wild-type *KCS1* and the *kcs1-*S537A,S646A mutant as described under Experimental Procedures. Strains were grown under conditions of nitrogen limitation prior to inositol polyphosphate extraction. As shown, InsP_7_ levels were significantly elevated in a strain with the *kcs1* allele mutated for Snf1-dependent phosphorylation sites. InsP levels were measured as the area under the curve of radiolabeled [H^3^] counts per min (CPMA^2^) for collected fractions. Mean results are shown with one standard deviation, and *p*-values were determined by Student’s *t* test. For comparison, results are included for a homozygous *snf1* deletion strain of the same filamentous Σ1278b background, with inositol polyphosphates extracted under identical conditions of nitrogen limitation. Significant differences in InsP_8_ abundance between the strains were not observed. The full set of inositol polyphosphate profiling data with [^3^H] counts for all fractions is available in File S2.

To consider the phenotypic importance of these Kcs1 phosphorylation sites, we profiled inositol polyphosphate levels in a filamentous strain of *S. cerevisiae* with alanine substitutions at Kcs1 Ser 537 and Ser 646. In yeast, Kcs1 principally generates the 5PP-InsP_5_ isoform of InsP_7_ (Fig. 3*A*) (62). For this study, we cloned the *KCS1* gene with its native promoter into a low-copy centromeric yeast shuttle vector. We mutated the codons for Ser 537 and Ser 646 to encode alanine, and the plasmid was introduced into a *kcs1* deletion strain in the filamentous Σ1278b background. We profiled inositol polyphosphate levels under conditions of nitrogen limitation conducive to pseudohyphal filamentation in the site-directed mutant strain and in *kcs1*Δ/Δ carrying wild-type *KCS1*. For comparison, we also profiled inositol polyphosphates in a homozygous strain of the Σ1278b background deleted for *SNF1*. Relative to the strain with wild-type *KCS1*, the *kcs1*-S537A,S646A mutant exhibited elevated levels of InsP_7_, comparable to levels observed in *snf1*Δ/Δ (Fig. 3*B*). We did not observe a significant difference in InsP_8_ levels between strains with wild-type *KCS1* and strains with the *kcs1*-S537A,S646A allele. The data indicate that Ser 537 and Ser 646 in Kcs1 contribute to the maintenance of wild-type levels of pyrophosphorylated InsP_7_.

### The kcs1-S537A,S646A mutant is impaired in pseudohyphal growth

**Figure 4:**
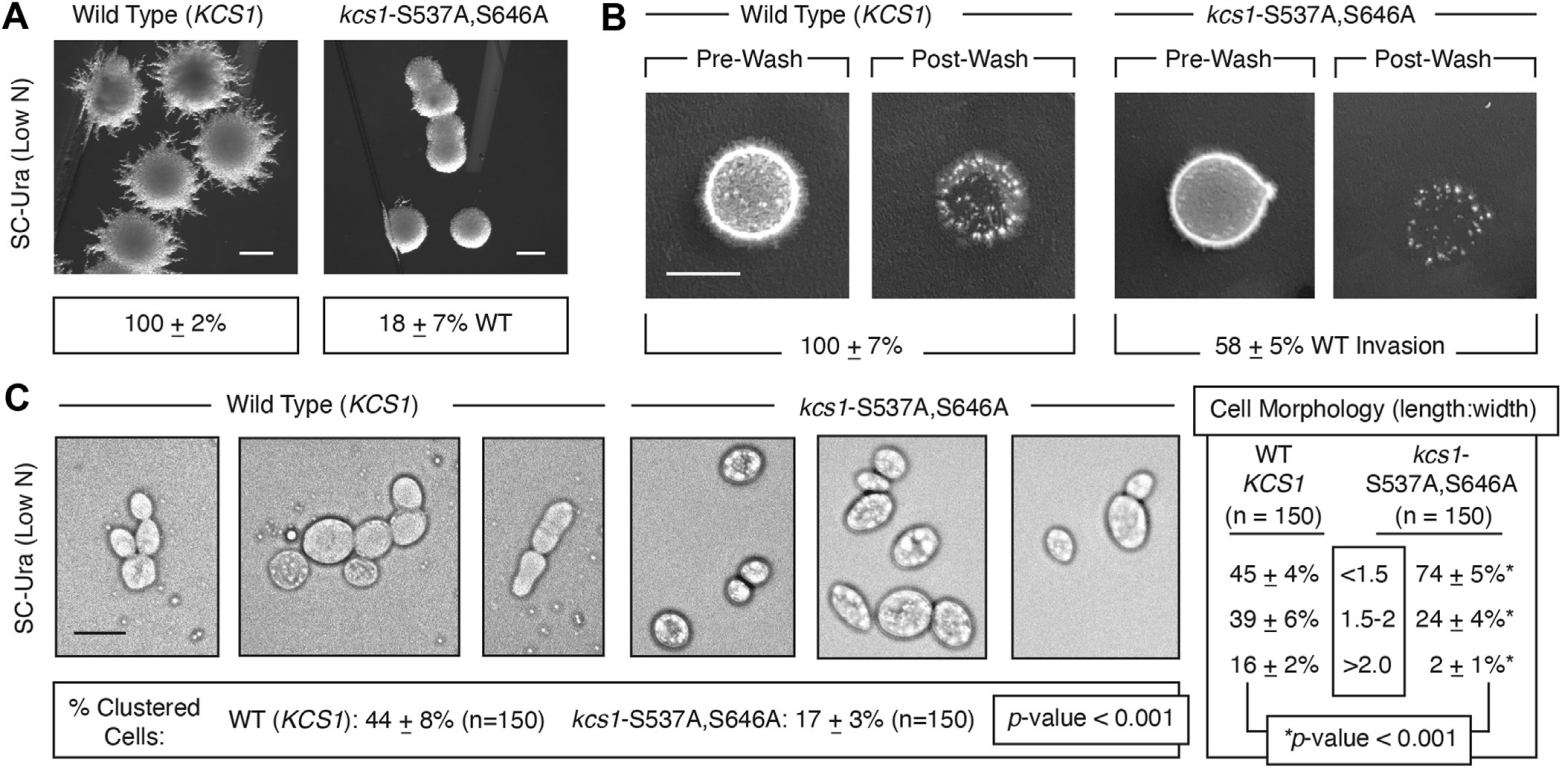
**Filamentous growth is decreased in the *kcs1*-S537A,S646A strain mutated for Snf1-dependent phosphorylation sites.***A*, surface filamentation of the control strain (*kcs1*Δ/Δ with a low-copy plasmid containing wild-type *KCS1* and its native promoter) and the isogenic strain with a plasmid containing *kcs1*-S537A,S646A is shown. Filamentation was quantified relative to the wild-type control strain using triplicate biological replicate cultures streaked on medium with low levels of ammonium sulfate. Percentages indicate mean values with standard deviation calculated as described. Scale bar, 2 mm. *B*, Agar invasion was assayed by washing plates under running water. Cultures of the strains were spotted on a growth medium with normal levels of nitrogen and glucose. The spotted cultures before and after washing were imaged, and the degree of invasion was quantified relative to the wild type. Mean values from three biological replicates are indicated with standard deviation. Scale bar, 1 cm. *C*, strains with wild-type *KCS1* and *kcs1*-S537A,S646A were cultured in liquid media with low levels of ammonium sulfate as a nitrogen source. The morphology of cultured cells was assessed by microscopy. The percentage of cells with indicated length-to-width ratios is presented as mean values with standard deviation from cell counts of 150 cells from each of two biological replicate cultures for both strains. Arrowheads indicate cells with a length-to-width ratio greater than 2. Scale bar, 5 μm.

Since *KCS1* is required for wild-type surface filamentation, agar invasion, and cell elongation, we assessed the phenotypic contributions of Kcs1 residues Ser 537 and Ser 646 to the yeast pseudohyphal growth response. Filamentous growth phenotypes for the *kcs1*Δ/Δ mutant are presented in Norman *et al.* (55). Under conditions of nitrogen limitation, the *kcs1-*S537A,S646A mutant shows significantly decreased surface filamentation relative to wild type (Fig. 4*A*). Invasive growth is assessed in filamentous strains of *S. cerevisiae* by growth of a spotted culture on normal medium and analysis of the degree to which colonies remain on the agar plate after washing off the surface of the plate (34). The *kcs1-*S537A,S646A mutant exhibits decreased agar invasion relative to wild type (Fig. 4*B*). Similarly, a culture of *kcs1-*S537A,S646A grown in liquid medium with low levels of the nitrogen source ammonium sulfate shows decreased cell elongation and diminished numbers of cell clusters relative to a wild-type strain grown under identical conditions (Fig. 4*C*).

### Transcriptional profiling identifies an extensive regulatory program affected in the kcs1-S537A,S646A strain

**Figure 5:**
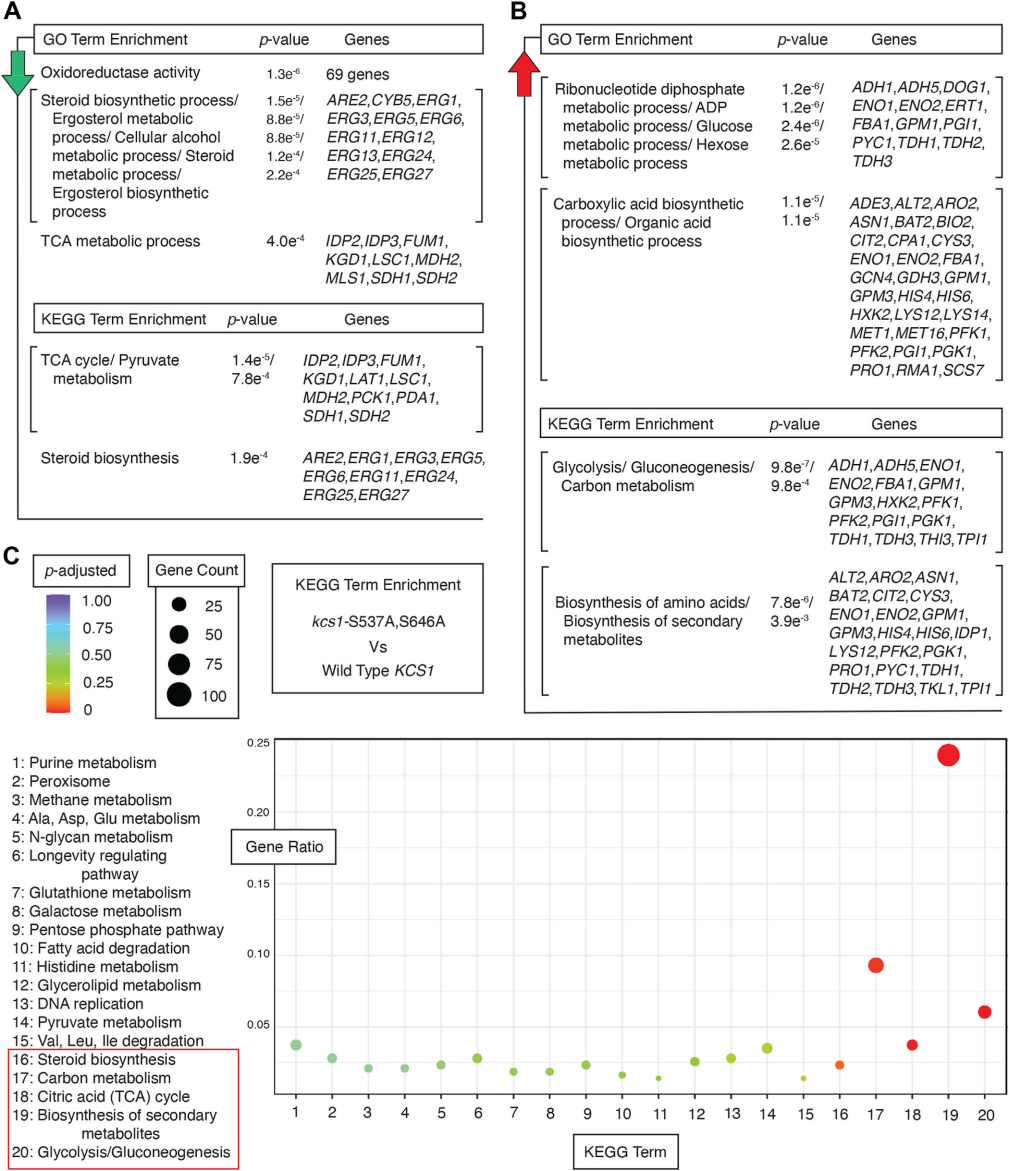
**Mutation of Kcs1 Ser 537 and Ser 646 affects an extensive transcriptional program.** GO terms and KEGG pathway terms enriched in the set of genes with differential transcript levels in *kcs1*-S537A,S646A are shown. For this analysis, genes were grouped into those exhibiting: *A*, decreased transcript levels in *kcs1*-S537A,S646A relative to wild type and *B*, those exhibiting increased levels. The *p*-value of the enrichment over a background gene set is indicated. *C*, *dot plot* presenting KEGG terms overrepresented among the genes differentially expressed in strains with *kcs1*-S537A,S646A *versus* strains with wild-type *KCS1*. KEGG terms are listed on the X-axis, with the key shown to the *left*. The Y-axis identifies the ratio of genes in the differential transcript dataset annotated with the indicated KEGG term as compared against genes annotated with the identical term in the genome as a whole. The size of the dot is proportional to the number of genes with the given KEGG term, and the Benjamini and Hochberg adjusted *p*-value of the enrichment is color-coded as shown.

Inositol polyphosphate signaling regulates transcript levels in several ways. The inositol kinase IP_6_K1 regulates histone modifications through association with chromatin and interaction with a histone lysine demethylase (85). Inositol pyrophosphates activate the class I histone deacetylase Rpd3L, regulating cell growth and the general stress response (86). InsP_7_ inhibits the mRNA decapping enzyme NUDT3 *in vitro*, and elevated cellular levels of InsP_7_ are associated with an increased abundance of NUDT3 mRNA substrates and increased numbers of processing bodies (87). Thus, pyrophosphorylated InsPs affect transcription and transcript levels. Consequently, we implemented RNA profiling to determine the importance of Snf1-dependent Kcs1p phosphorylation sites Ser 537 and Ser 646 on global transcript levels. We cultured three biological replicates each of *kcs1*-S537A,S646A, and its parent strain with wild-type *KCS1* to steady-state in low-nitrogen media for RNA extraction and subsequent high-throughput sequencing. Results are presented in Figure 5, and quality/summary statistics are presented as Supporting Information (Figs. S3–S5).

As compared to the wild type, 1201 transcript levels are affected in the *kcs1*-S537A,S646A mutant, encompassing 500 transcripts with increased abundance and 701 transcripts with decreased levels. The set of transcripts decreased in *kcs1*-S537A,S646A relative to wild type is notably enriched for genes annotated with Gene Ontology (GO) Biological Process terms of oxidoreductase activity, steroid biosynthetic processes, and the tricarboxylic acid (TCA) cycle (Fig. 5*A*). A similar set of genes involved in the TCA cycle and steroid biosynthesis were identified by enrichment analysis of KEGG terms for this gene set.

The set of genes with increased transcript levels in the *kcs1*-S537A,S646A mutant relative to wild type is enriched for GO Biological Process terms associated with ADP and glucose metabolism (Fig. 5*B*). Genes encoding proteins functioning in carboxylic acid biosynthesis are also enriched in this dataset. Similar terms from the KEGG database are associated with these genes, identifying processes of glycolysis, gluconeogenesis, and amino acid biosynthesis. The total set of genes altered in *kcs1*-S537A,S646A is enriched for KEGG terms presented as a dot plot in Figure 5*C*. Terms with significantly low adjusted *p*-values are boxed. Additional gene enrichment analyses of these data sets for GO biological process and molecular function terms are provided in Figs. S6 and S7.

### Growth of kcs1-S537A,S646A is affected on sucrose-containing media containing antimycin A

**Figure 6:**
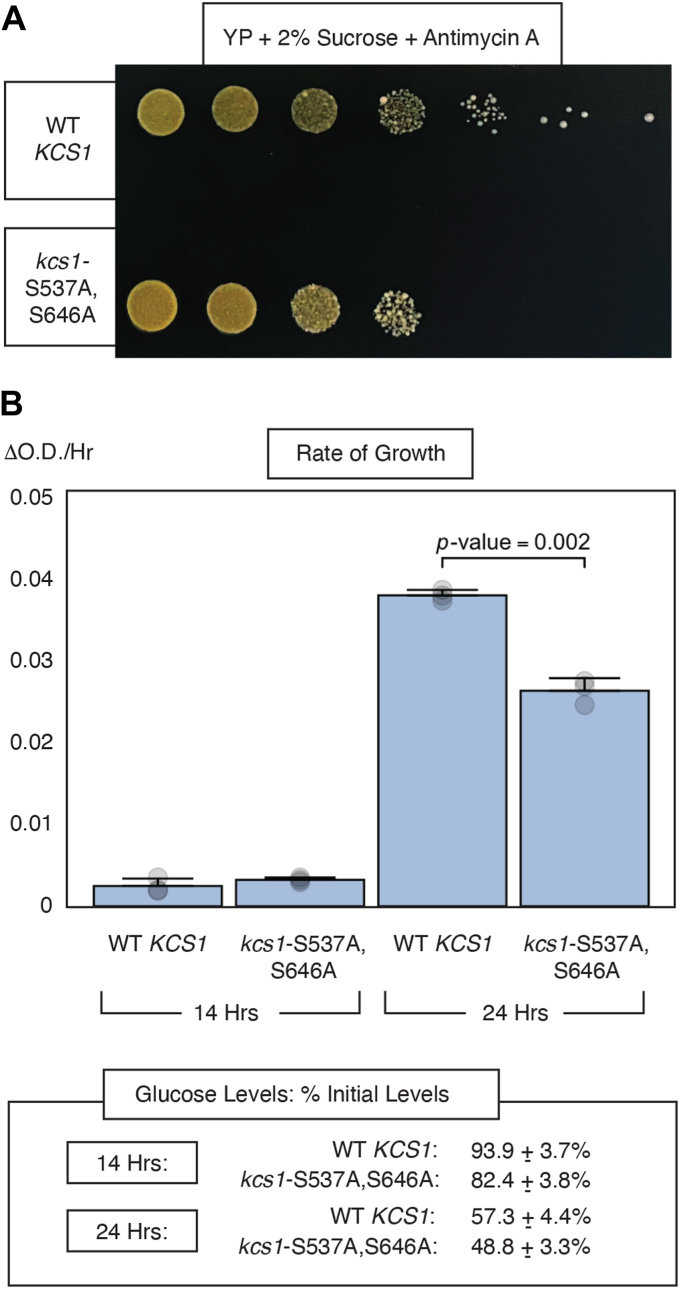
**Growth in sucrose is affected by mutation of Ser 537 and Ser 646 in Kcs1.***A*, cultures with wild-type *KCS1* and *kcs1-*S537A,S646A were grown overnight in normal media, and three-fold serial dilutions of the cultures were subsequently spotted onto plates with YP medium containing sucrose and antimycin A. Plates were imaged after 3 days growth at 30 °C, and a representative image is shown here. *Top row*: wild-type *KCS1*; *bottom row*: *kcs1*-S537A,S646A. *B*, the bar graph indicates rate of growth for strains with wild-type *KCS1* and the *kcs1*-S537A,S646A mutant at 14 h and 24 h post inoculation in standard SC -Ura media with 2% glucose. Data are presented as mean rate of growth (n = 3) with error bars of one standard deviation. Individual data points are included, and the *p*-value was determined by student’s *t* test. Glucose levels are shown as a mean percentage of starting glucose concentration with an error of one standard deviation. Little additional growth of either strain was observed after 24 h post inoculation.

Snf1 is activated under conditions of glucose limitation, and *SNF1* is required for the adaptation of yeast cells to growth on media with a non-fermentable carbon source (13). Yeast strains grown on carbon sources that do not support fermentation typically derepress a suite of genes, including TCA cycle genes and other genes involved in aerobic respiration. This activated gene set can enable yeast cells grown on non-fermentable sucrose to acquire more robust respiratory systems than genetically identical cells grown on glucose, potentially masking *snf1*-associated phenotypes (13, 88). Phenotypes stemming from defective Snf1 signaling can, therefore, be effectively scored on glucose-limited or non-fermentable media under anaerobic conditions (88). To consider sucrose fermentation phenotypes for *kcs1*-S537A,S646A, we spotted serial dilutions of the *kcs1*-S537A,S646A mutant and a control culture with wild-type *KCS1* on media with sucrose as a carbon source supplemented with the electron transport chain inhibitor antimycin A. As indicated in Figure 6*A*, the *kcs1*-S537A,S646A mutant exhibited a moderate growth defect under these conditions. Strains deleted for *SNF1* do not exhibit a growth defect on media with normal levels of glucose; however, Snf1 signaling is important for yeast cell growth as glucose levels become limiting. Here, we assessed the growth rate of strains with wild-type *KCS1 versus* strains carrying the *kcs1*-S537A,S646A mutation. In liquid culture, the growth rate of these strains in standard glucose-containing media did not differ significantly at 14 h post inoculation; the rate of change of growth was low for both strains. At 24 h, the growth rate of both strains had increased, but the rate of change in growth for the *kcs1*-S537A,S646A strain was significantly less than the rate observed for the strain with wild type *KCS1* (Fig. 6*B*). At 14 h, glucose levels in the wild-type and *kcs1-*S537A,S646A cultures were roughly 82% and 94%, respectively, of levels observed initially upon inoculation. At 24 h, glucose levels had dropped to approximately 57% and 49% of starting levels in the strain with wild-type *KCS1* and *kcs1*-S537A,S646A, respectively (Fig. 6*B*).

## Discussion

Here, we address the regulation of InsP signaling by Snf1/AMPK and the phenotypic effects of this relationship. The inositol pyrophosphate kinase Kcs1 is phosphorylated in a Snf1-dependent manner *in vivo* at Ser 537 and Ser 646. Kcs1 is phosphorylated *in vitro* by the Snf1 kinase complex, principally at the same serine residues. These Snf1-dependent phosphorylation sites are required for wild-type levels of pyrophopshorylated InsP_7_, with levels elevated in a *kcs1* mutant encoding alanine at Ser 537 and Ser 646. Pseudohyphal filamentation is decreased in *kcs1*-S537A,S646A, with corresponding decreases in cell elongation and invasive growth. An extensive transcriptional program is perturbed in the *kcs1*-S537A,S646A mutant, encompassing genes involved in steroid biosynthesis, glycolysis, gluconeogenesis, and the TCA cycle. Growth of *kcs1*-S537A,S646A is decreased relative to wild type on non-fermentable media with sucrose and the electron transport chain inhibitor antimycin A.

These data identify Kcs1 as a substrate and effector of Snf1/AMPK signaling. In regards to the latter point, *kcs1*-S537A,S646A phenotypes resemble *snf1* loss-of-function phenotypes, as would be expected for a gene acting downstream of *SNF1*. It is notable that *kcs1*-S537A,S646A phenotypes are predominantly less severe than those seen in *snf1*Δ/Δ. Pseudohyphal filamentation is diminished in *kcs1*-S537A,S646A, but not completely lost as it is for a *snf1*Δ/Δ mutant in a filamentous background. The growth of *kcs1*-S537A,S646A is affected on media with sucrose and antimycin A, but not to the degree observed in previously published work for *snf1*Δ/Δ (23). A partially overlapping set of genes is differentially expressed between the *snf1*Δ/Δ and *kcs1*-S537A,S646A mutants: of 59 regulatory or transport-related genes differentially expressed in *snf1*Δ cells (31, 89), 22 were also differentially expressed in the *kcs1*-S537A,S646A mutant. The Snf1 signaling network is extensive; many Snf1 signaling outputs are necessary to regulate catabolism and glucose derepression. Our data indicate that Snf1-mediated phosphorylation of Kcs1 is one newly identified mechanism in a regulatory network through which Snf1 signaling is accomplished.

The identification of Snf1-mediated phosphorylation of Kcs1 addresses a mechanistic gap in understanding the upstream regulatory pathways that control InsP biosynthesis and signaling. InsP_7_ levels are elevated in a *snf1*Δ/Δ strain, and we also observe elevated InsP_7_ levels in *kcs1*-S537A,S646A. The regulatory relationship between AMPK and InsP signaling, however, is complex and extends beyond the AMPK-dependent phosphorylation of Kcs1. In hypothalamic lysates of mouse brain, glucose availability results in the phosphorylation of inositol polyphosphate multikinase (IPMK), the metazoan ortholog of yeast Arg82, and its binding to AMPK; this binding inhibits AMPK phosphorylation and decreases AMPK catalytic activity (74). Metazoan *IP6K1* is orthologous to yeast *KCS1*. IP6K1 is reportedly antagonistic to AMPK, as overexpression of *IP6K1* in HEK293 cells decreases phosphorylation and activity of AMPK (90). From *in vitro* studies, InsP_6_, a substrate of IP6K1, stimulates phosphorylation of AMPK by the upstream AMPK-activating Liver Kinase B1 (LKB1) (90). IP6K1 converts InsP_6_ to InsP_7_, potentially neutralizing InsP_6_-mediated AMPK activation through LKB1. Conversely, InsP signaling can stimulate AMPK activation under conditions of extreme stress. Data suggest that in mouse embryonic fibroblast cells grown in low glucose followed by treatment with the AMPK activator and the adenosine analog AICAR, which collectively mimics extreme stress, LKB1 is phosphorylated, exits the nucleus, and activates AMPK (91). Thus, several lines of evidence indicate that InsP signaling regulates AMPK activity in response to the nutritional state of the cell. The data here identify a mechanism whereby AMPK regulates InsP levels and particularly the levels of inositol pyrophosphate InsP_7_. The results highlight a bidirectional regulatory relationship between AMPK and InsP signaling, with IPMK inhibiting AMPK under conditions of glucose availability and Snf1 phosphorylating Kcs1 under conditions of nutrient limitation.

InsP_6_ kinase functionality is conserved in eukaryotes from yeast to humans; however, the sequences of these proteins vary dramatically. Yeast Kcs1 is 1050 amino acids in length, while human *IP6K* genes encode proteins from 410 (IP6K3) to 441 amino acids (IP6K1). IP6K1 protein in mice is 433 amino acids in length, and the *Drosophila melanogaster* ortholog IP6K-PB is 672 amino acids. Consequently, there is little conservation of primary sequence blocks in Kcs1 orthologs readily observable from amino acid alignment outside of the kinase domain (roughly amino acids 759–956 in Kcs1). A diagram of Kcs1 protein domains and the relative locations of Ser 537 and 646 phosphorylation sites is provided in Fig. S1*A*. Both the Ser 537 and 646 phosphorylation sites are conserved across sequenced non-filamentous and filamentous *S. cerevisiae* strains.

The Kcs1 Ser 537 and 646 phosphorylation sites are required for pseudohyphal growth, with Snf1p-mediated phosphorylation of Kcs1 potentially regulating signaling through several avenues. Pyrophosphorylated InsPs can bind target proteins for allosteric regulation of activity (92, 93, 94). Wu and colleagues (73) used chemical analogs of InsP_6_ and InsP_7_ as affinity reagents to identify binding substrates; however, proteins providing a clear link to pseudohyphal growth were not apparent in the set of identified targets. InsP_7_ species have been found to pyrophosphorylate target proteins independent of InsP kinase activity (95, 96). Additionally, InsP species can modulate transcription through chromatin remodeling, as the Arg82 InsP kinase is required for wild-type activity of the SWI/SNF and INO80 chromatin remodeling complexes (97). Beon *et al.* (98) found that mammalian IPMK directly binds to the SWI/SNF complex components SMARCB1, BRG1, and SMARCC1, affecting promoter targeting and downstream processes in mouse embryonic stem cells.

Kcs1 is an extensively phosphorylated protein (78, 79), and additional upstream signaling pathways may regulate Kcs1 activity. As indicated in Figure 1*B*, residual phosphorylation of Ser 646 is present in a strain with a kinase-defective allele of *SNF1*, supporting the notion that additional signaling systems may act on Kcs1. Fig. S1*B* indicates the results of predictive tools for the identification of matched kinase substrate consensus motifs and phosphorylated residues, and the analysis is consistent with mass spectrometry data showing the phosphorylation of many residues in Kcs1. Certainly, pyrophosphorylated InsP species are critical signaling messengers in the response to cellular nutrient and energy status, and, as such, multiple nutrient-responsive signaling networks likely act to control the respective levels of InsP species in the inositol polyphosphate biosynthesis pathway. In this context, this work is a step towards understanding the upstream signaling network feeding into InsP signaling, identifying the Kcs1 InsP kinase as an AMPK signaling substrate and a regulatory node coordinating processes of cell metabolism and growth with cell energetics and nutrient availability.

## Experimental procedures

### Yeast strains, plasmids, and culture conditions

Yeast strains (Table S1) and plasmids (Table S2) used in this study are indicated. Yeast strains are derived from the filamentous Σ1278b genetic background (34, 99). Yeast cells were cultured according to standard protocols (100) as described. We prepared low-nitrogen synthetic low-ammonia dextrose (SLAD) media as follows: 0.17% yeast nitrogen base without amino acids and without ammonium sulfate, 2% glucose, 50 μM ammonium sulfate, and appropriate amino acids to complement auxotrophy. YPD media contained 2% peptone, 1% yeast extract, 2% glucose, and 2% agar, as needed. SC media and derivatives were purchased from Sunrise Science Products. The formulation for SC media is as follows: 0.67% yeast nitrogen base, 2% glucose, 0.5% ammonium sulfate (normal nitrogen levels) or 50 μM ammonium sulfate (low nitrogen levels), 0.2% of the appropriate amino acid dropout mix, and 2% agar as needed. For inositol polyphosphate profiling, yeast cultures were grown in media lacking inositol (0.17% yeast nitrogen base without amino acids, ammonium sulfate, and inositol; 2% glucose; 0.5% ammonium sulfate; and appropriate amino acids) or in SLAD-inositol media (0.17% yeast nitrogen base without amino acids, ammonium sulfate, and inositol; 2% glucose; 50 μM ammonium sulfate; and required amino acids).

### Mass spectrometry of Snf1-dependent Kcs1 phosphorylation

Mass spectrometry was performed largely as described previously (51) through the University of Michigan Proteomics Resource Facility. For Stable Isotope Labeling by Amino Acids in Culture (SILAC), yeast strains with deletions of *ARG4* and *LYS1* were used, enabling auxotrophies for the uptake of arginine and lysine amino acids with different stable isotopes of carbon, nitrogen, and/or hydrogen (deuterium) (Cambridge Isotope Laboratories, Inc). Strains were cultured in triplicate under indicated growth conditions with the light (natural) versions of L-lysine and L-arginine used to label wild-type yeast and heavy (Lys-8/Arg-10) L-lysine and L-arginine labeling the *snf1* kinase-dead allele strain. For the analysis of phosphorylation under conditions of low nitrogen, light lysine, and arginine were incorporated into media with normal levels of nitrogen, and heavy lysine and arginine were incorporated into low-nitrogen media. To achieve sufficient labeling, strains were cultured overnight, diluted in SILAC media, and incubated at 30 °C for approximately 10 doublings. Labeled cultures were pelleted and frozen prior to protein extraction using a bead beater and lysis buffer (50 mM Tris buffer [pH 8.2], 8 M urea, protease inhibitors [Roche], and phosphatase inhibitors). Protein samples were digested with trypsin at 37 °C overnight. Peptide mixtures were separated by strong cation exchange into fractions, and the fractions were enriched for phosphopeptides using zirconium dioxide (ZrO_2_)-based columns. The ZrO_2_ eluate and the flow-through of each strong cation exchange column were analyzed by nanoLC-tandem mass spectrometry. Samples were separated using a column for introduction *via* an electrospray device to an LTQ-Orbitrap XL hybrid-type mass spectrometer. The resulting data were quantified using Maxquant and the Mascot search engine. Yeast open reading frames from the *Saccharomyces* Genome Database were used for peptide identification.

### Expression and purification of Kcs1 fragments

PCR products encoding amino acids 1 to 250, amino acids 509 to 780 and amino acids 755 to 1050 of Kcs1 were cloned into vector pMAL-C2. The PCR product encoding Kcs1 amino acids 244 to 556 was cloned into pGEX-4T. Proteins were expressed as maltose binding protein (MBP) or glutathione S-transferase (GST) fusions, with a sequence encoding the MBP- and GST-tags fused immediately upstream of the *KCS1* sequence. Kcs1 fusion proteins with GST or MBP at the amino terminus were purified with amylose or glutathione agarose beads. The GST-Mig1 fusion protein was purified similarly.

### Purification of active Snf1 kinase complex

*E. coli* expressing activated Snf1 complexes was a kind gift from Martin C. Schmidt (University of Pittsburgh) (84). 100 ml of log-phase cells grown in 2× yeast extract tryptone glucose (YPD) medium was induced for 3 h by the addition of 0.4 mM IPTG. Cells were pelleted and resuspended in 10 ml of 25 mM Tris-HCl, 200 mM NaCl, 10 mM imidazole, 5 mM β-mercaptoethanol with Roche protease inhibitors and 0.5 mM PMSF, pH 7.4. Lysozyme was added at 1 mg/ml on ice for 20 min followed by sonication. Lysates were centrifuged for 10 min at 12,000*g*, and 200 μl of washed Talon beads were added to the supernatant for 1 h with rocking at 4 °C. The beads were then washed three times with 1 ml lysis buffer, and Snf1 complexes were eluted with 200 mM imidazole in 0.5 ml of lysis buffer. 50% glycerol was added to the eluate prior to storage at −20 °C.

### *In vitro* kinase assays

Kinase assays were performed in 20 μl volumes of buffer containing 20 mM HEPES, 100 mM NaCl, 5 mM MgCl_2_, and 1 mM DTT, with approximately 2 μg of fusion protein substrates and 3 μl of purified Snf1 kinase complex. Reactions were initiated by the addition of ^32^P-ATP (final concentration of 1 μM, 0.1 μCi) and incubated for 30 min at room temperature. For mass spectrometry, reactions were performed as described except that 4 μg of Kcs1 fragment 3 (encoding AA 509–780) was added plus or minus 1 mM cold ATP. Mass spectrometry was performed at the University of Michigan Proteomics and Peptide Synthesis core.

### Inositol polyphosphate profiling

Analysis of InsP levels was conducted as described previously (55, 101) with modifications. Yeast cultures (5 ml) were grown overnight with shaking (250 rpm) at 30 °C until fully saturated (roughly 32–48 h). Subsequently 5 μl of the saturated culture was added to 5 ml of growth media lacking inositol. The media was supplemented with 25 μl of *myo*[1,2-^3^H] inositol 1 mCi/ml 30 Ci/mmol (American Radiolabeled Chemicals, Inc, catalog number ART261A), and we achieved inositol radiolabeling by growing the culture with shaking (250 rpm) to an OD_600_ of 0.9 (roughly 24 h). Cells were harvested, washed with water, and the resulting cell pellet was suspended in low-nitrogen media lacking inositol. The culture was grown for three cell doublings at 30 °C with shaking at 250 rpm. Cells were harvested and frozen at −80 °C for later use. InsPs were extracted by suspending the thawed pellet in 300 μl of 1M perchloric acid with 3 mM EDTA; the suspension was treated in a bead beater for 5 min at 4 °C. Samples were centrifuged, and the resulting supernatant was retained and neutralized with the addition of 1M potassium carbonate and 3 mM EDTA to achieve a roughly neutral pH of 6.0 to 8.0. The mixture was incubated on ice for 2 h. Subsequently, the mixture was centrifuged, and the supernatant was analyzed by HPLC using a 5 μm SAX cartridge column (125 × 4.6 mm). InsPs were eluted from the column with a gradient from mixing buffer A (1 mM EDTA) and buffer B (1.3 M (NH_4_)_2_HPO_4_, 1 mM EDTA, pH 3.8). The gradient was as follows: 0 to 5 min with 0% buffer B (100% buffer A); 5 to 10 min with 0 to 10% buffer B; 10 to 90 min with 10 to 100% buffer B; 90 to 100 min with 100% buffer B; 100 to 101 min with 0% buffer B (100% buffer A); 101 to 110 min with 0% buffer B (100% buffer A). The gradient was run at a flow rate of 1 ml/min, and 1 ml fractions were collected every minute for the first 90 min. Each fraction was mixed with 4 ml of Ultima-Flo AP liquid scintillation cocktail (Revvity Life Science and Diagnostics, Inc), and radiolabel was quantified using a scintillation counter (File S2). InsP_7_ levels were calculated as the area under a peak above background for relevant fractions, and data were normalized by subtracting the lowest radiolabel count value for the fractions. InsP_7_ values are indicated as the mean with error bars indicating one standard deviation. Comparative significance was determined by student’s *t* test.

### Filamentous growth assays

Surface-spread filamentation, invasive growth, and cell morphology were assayed according to standard protocols (34, 37, 102, 103). Surface filamentation and invasive growth were imaged and quantified using Image J (104) as described previously (55, 103, 105). To assess surface-spread filamentation, yeast cultures were incubated on a solid SC-Ura medium with low levels of ammonium sulfate at a density of roughly 50 colonies per plate. Growth plates were cultured at 30 °C until wild-type colonies exhibited surface-spread pseudohyphal filaments. We quantified the degree of surface filamentation using Image J. For these studies, we determined the circumference of a defined area of a colony, encompassing pseudohyphal filaments at the periphery of the colony, and compare this value against the circumference of the same defined area of a wild-type colony. The degree of surface filamentation is calculated relative to wild-type (set as 100%) from five independent colonies on multiple plates streaked with biological replicates of the starting culture. To determine agar invasion, we spotted a 5 μl aliquot of the culture to be tested onto a plate with standard growth medium (normal levels of nitrogen) for incubation at 30 °C until the cultures exhibit dense growth (roughly 4–5 days). Plates were photographed, and the agar surface was washed under a gentle stream of water to remove non-invasive cells. We subsequently photographed the washed plate and used Image J to quantify the degree of invasive growth as mean pixel intensity of the washed spot relative to the corresponding mean pixel intensity of the unwashed spotted culture. Identical areas were used for each pixel calculation, and we assayed spots from three biological replicate cultures for each tested strain. Values are presented as a percentage of wild-type invasion with standard deviation indicated. To determine cell morphology and cell-cell adhesion, liquid cultures of the yeast strains were grown in low-nitrogen media. Cells were cultured for approximately 24 h at 30 °C with shaking at 250 rpm. After incubation, cells were imaged by differential interference contrast microscopy. We subsequently measured length:width dimensions for 150 cells from each culture, and the experiment was repeated with three biological replicate cultures for each strain tested.

### Transcriptional profiling

We inoculated three single colonies each of a control strain (SSY128: *kcs1*Δ/Δ with pRS416-*KCS1*) and *kcs1* phosphodefective strain (SSY146: *kcs1*Δ/Δ with pRS416-*kcs1*-S537A,S646A) in 5 ml of SC -Ura media at 30 °C with shaking (250 rpm). Cultures were grown to saturation and were subsequently diluted in 50 ml of low-nitrogen media to an absorbance (optical density at 600 nm) of 0.05. Cultures were grown at 30 °C with shaking for roughly 18 to 20 h to an optical density of approximately 0.5 at 600 nm. We subsequently harvested cells from each culture and stored the cell pellets at −80 °C. We isolated RNA from the frozen pellets using the RiboPure Yeast Kit (Thermo Fisher Scientific). RNA samples were treated with DNase, and RNA concentrations were determined using the NanoDrop microvolume spectrophotometer.

RNA quality control, sequencing, and analysis were performed by Novogene. RNA integrity was assessed using the RNA Nano 6000 Assay Kit of the Bioanalyzer 2100 system (Agilent Technologies, CA, USA). Libraries were prepared for sequencing as follows. Poly(A)-mRNA was purified from total RNA using poly(T) oligo-attached magnetic beads. Fragmentation was carried out using divalent cations under elevated temperature in the First Strand Synthesis Reaction Buffer (5×). First-strand cDNA was synthesized using random hexamer primers and M-MuLV Reverse Transcriptase (RNase H-). Second-strand cDNA synthesis was performed using DNA Pol I and RNase H. Remaining overhangs were converted into blunt ends through exonuclease/polymerase activities. The 3′-ends of DNA fragments were adenylated, and adapter sequences with hairpin loops were ligated to the DNA. cDNA fragments of roughly 370 to 420 bp were preferentially selected using the AMPure XP system (Beckman Coulter). PCR was performed using Phusion High-Fidelity DNA Polymerase, universal PCR primers, and Index (X) primers. PCR products were purified, and library quality was assessed using the Agilent Bioanalyzer 2100 system.

Index-coded samples were clustered on a cBot Cluster Generation System using the TruSeq PE Cluster kit V3-cBot-HS according to manufacturer instructions. The library preparations were sequenced on an Illumina Novaseq platform for 150-bp paired-end reads. Raw data reads of fastq format were processed through in-house perl scripts (Novagene) to remove reads with adapter sequences and low-quality sequences. Reads were subsequently mapped to the yeast genome using hisat v2.0.5 (Novogene). Transcript levels were quantified per gene using featureCounts v1.5.0-p3, and the expected number of fragments per kilobase of transcript per million bp sequenced (FPKM) was calculated to estimate gene expression levels. Differential expression between samples was determined using the DESeq2 R package (1.20.0). The resulting *p*-values were adjusted using Benjamini and Hochberg’s approach for controlling the false discovery rate. Genes with an adjusted *p* value ≥0.05 by analysis with DSeq2 were considered to be differentially expressed.

Gene Ontology (GO) enrichment was assessed using the clusterProfiler R package, and GO terms with a corrected *p*-value of < 0.05 were selected as being significantly enriched. The clusterProfiler R package was also used to identify statistically enriched gene sets from pathway data in the Kyoto Encyclopedia of Genes and Genomes (KEGG) database. Enrichment of gene sets in the data was further considered by Gene Set Enrichment Analysis (GSEA) using a publicly available version of the GSEA software from the Broad Institute with GO and KEGG datasets.

### Kcs1-S537A,S646A growth phenotypes

Growth defects associated with inhibited *snf1* function were assessed by growing wild-type and mutant strains on YP media (2% yeast extract, 1% peptone, and 2% agar) with 2% sucrose and antimycin A to a final concentration of 1 μg/ml. Growth rates for yeast strains were determined on SC-Ura media with 2% glucose as a carbon source for 24 h incubation at 30° C with shaking. Growth rates of *kcs1* deletion strains carrying wild-type *KCS1* and the *kcs1-*S537A,S646A mutant were calculated as the slope (increase in culture optical density over time) from the previous time point. Mean growth rate was calculated from triplicate biological replicates, and the error bar indicates one standard deviation. Glucose levels in liquid cultures were measured using the Glucose-Glo assay kit (Promega). Glucose levels are shown as a mean percentage of starting glucose concentration with error bars indicating one standard deviation.

## Data availability

Study data are available in supporting information. Transcription profiling data are available in the Gene Expression Omnibus (accession number GSE246711).

## Supporting information

This article contains supporting information.

## Conflict of interest

The authors declare that they have no known competing financial interests or personal relationships that could have appeared to influence the work reported in this paper.
