# Supplementary material for: The yeast AMP-activated protein kinase Snf1 phosphorylates the inositol polyphosphate kinase Kcs1
Source: J Biol Chem. 2024 Jan 13;300(2):105657. doi: 10.1016/j.jbc.2024.105657 (PMC10851228; doi:10.1016/j.jbc.2024.105657)
Supplement: Supporting information [file mmc3.pdf]

The yeast AMP-activated protein kinase Snf1 phosphorylates the inositol polyphosphate kinase Kcs1

Sham Sunder, Joshua S. Bauman, Stuart J. Decker, Alexandra R. Lifton, and Anuj Kumar

Department of Molecular, Cellular, and Developmental Biology, University of Michigan, Ann Arbor, MI 48109, USA

Corresponding Author:

Anuj Kumar, Professor, Department of Molecular, Cellular, and Developmental Biology, University of Michigan, 3210 Biological Sciences Building, 1105 N. University Avenue, Ann Arbor, MI 48109

Phone: 734-647-8060

Email: anujk@umich.edu

Supporting Information includes:

- Figure S1 to Figure S7
- Table S1. List of strains used in this study
- Table S2. List of plasmids used in this study
- File S1. Excel spreadsheet of Kcs1 phosphorylation in filamentous Σ1278b under conditions of low nitrogen (.xlsx)
- File S2. Excel spreadsheet of inositol polyphosphate profiles for filamentous yeast Σ1278b carrying wild-type *KCS1* or *kcs1*-S537A,S646A under conditions of low nitrogen (.xlsx)

A

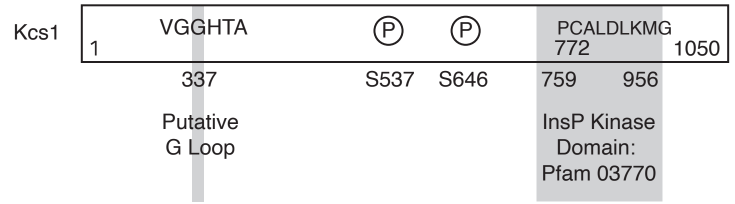

B

NetPhos 3.1 Prediction of Kinases that Phosphorylate Kcs1:

Residue	Location	Sequence	Score
S	537	...KRRD**S**GSTM...	0.995 (unspecified)<br>0.871 (PKA)
S	646	...DGSH**S**VMDL...	0.994 (unspecified)

PhosphoSVM Prediction of Phosphorylated Residues in Kcs1:

Rank	Location	Sequence	Score
2	537	...KRRD**S**GSTMINT...	0.892
27	646	...ALDGSH**S**VMDLK...	0.602

C AMPK Phosphorylation Substrate Motif:

	-6	-5	-4	-3		-1	P	+1		+3	+4
	β	ϕ	β	β	X	X	S/T	X	X	α	ϕ
Ser 537	**K**	**L**	**K**	**R**	R	D	**S**	G	S	T	**M**
Ser 646	A	**L**	D	G	S	H	**S**	V	M	**D**	**L**

Hydrophobic Residue:	ϕ	A, F, G, I, L, M, P, V, Y
Basic Residue:	β	H, K, R
Acidic Residue:	α	D, E

Figure S1. The Kcs1p Ser 537 and Ser 646 phosphorylation sites. *A*, A diagram of Kcs1 is shown, with its putative G loop and inositol kinase domain indicated. *B*, We analyzed the Kcs1 sequence for kinase substrate motifs using the server NetPhos 3.1 Results are shown for Ser 537 and 646. The strength of the score increases as it approaches 1. The AMPK consensus motif is not incorporated in NetPhos 3.1. Both residues are predicted to be phosphorylated by an unspecified kinase, with S537 as a possible PKA substrate as well. The online phosphorylation site prediction tool PhosphoSVM was used to predict phosphorylation sites in Kcs1. Both Ser 537 and 646 were predicted as phosphorylated residues; the server predicts phosphorylation sites without predicting a kinase responsible for the phosphorylation. Location indicates the amino acid position of the predicted phosphorylation site, and the strength of the prediction increases as the score approaches 1. *C* , The consensus AMPK phosphorylation substrate motif is indicated with hydrophobic amino acids, basic amino acids, and acidic residues indicated. Sequences flanking Kcs1 Ser 537 and Ser 646 are shown, and matching amino acids are shown in bold. The key hydrophobic residues conserved at the -5 and +4 positions are boxed in dashed lines. L or M is typically found at the -5 position, and L is typically observed at the +4 position, although I, M, V, or F has also been observed in AMPK substrates. The other positions indicated in the consensus motif are less stringently conserved.

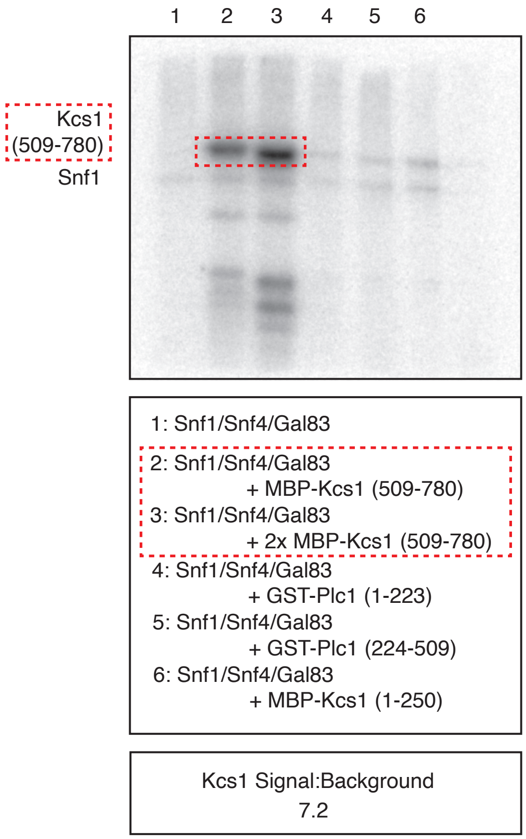

Figure S2. Purified Snf1 kinase complex phosphorylates Kcs1. Results from *in vitro* kinase assays are shown, indicating selective Snf1 phosphorylation of Kcs1 fragment 3 encoding amino acids 509-780 (Lanes 2 and 3). Twice the concentration of MBP-Kcs1 (509-780) was used in Lane 3. The band in Lanes 2 and 3 corresponding to the full-length product is shown boxed in red. Purified GST fusions to phospholipase C fragments (Plc1) were also tested for Snf1 phosphorylation; however, only a faint band was observed. Signal-to-background ratios for Kcs1 fragment phosphorylation by Snf1 is indicated.

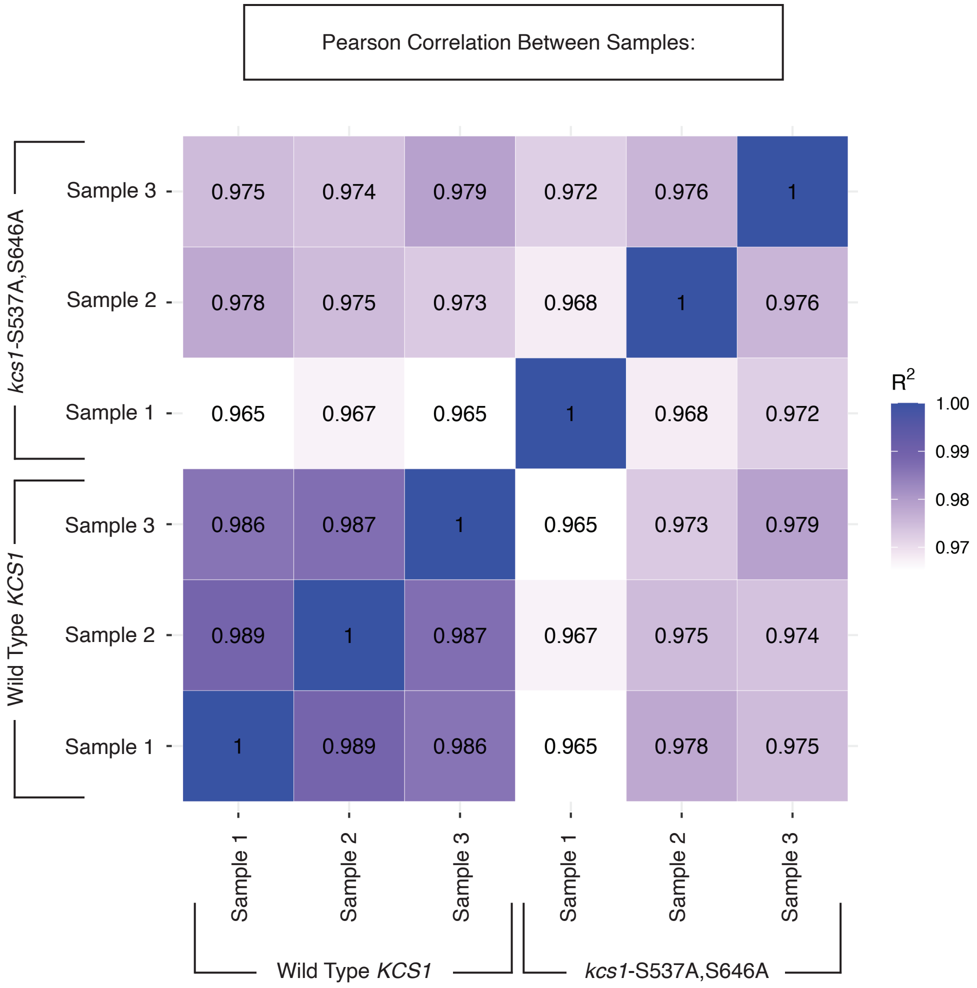

Figure S3. Correlation of transcript levels between biological replicates and strains used for RNA sequencing. Pearson correlation coefficients between samples and strains are indicated, and the correlation coefficients are color-coded as shown.

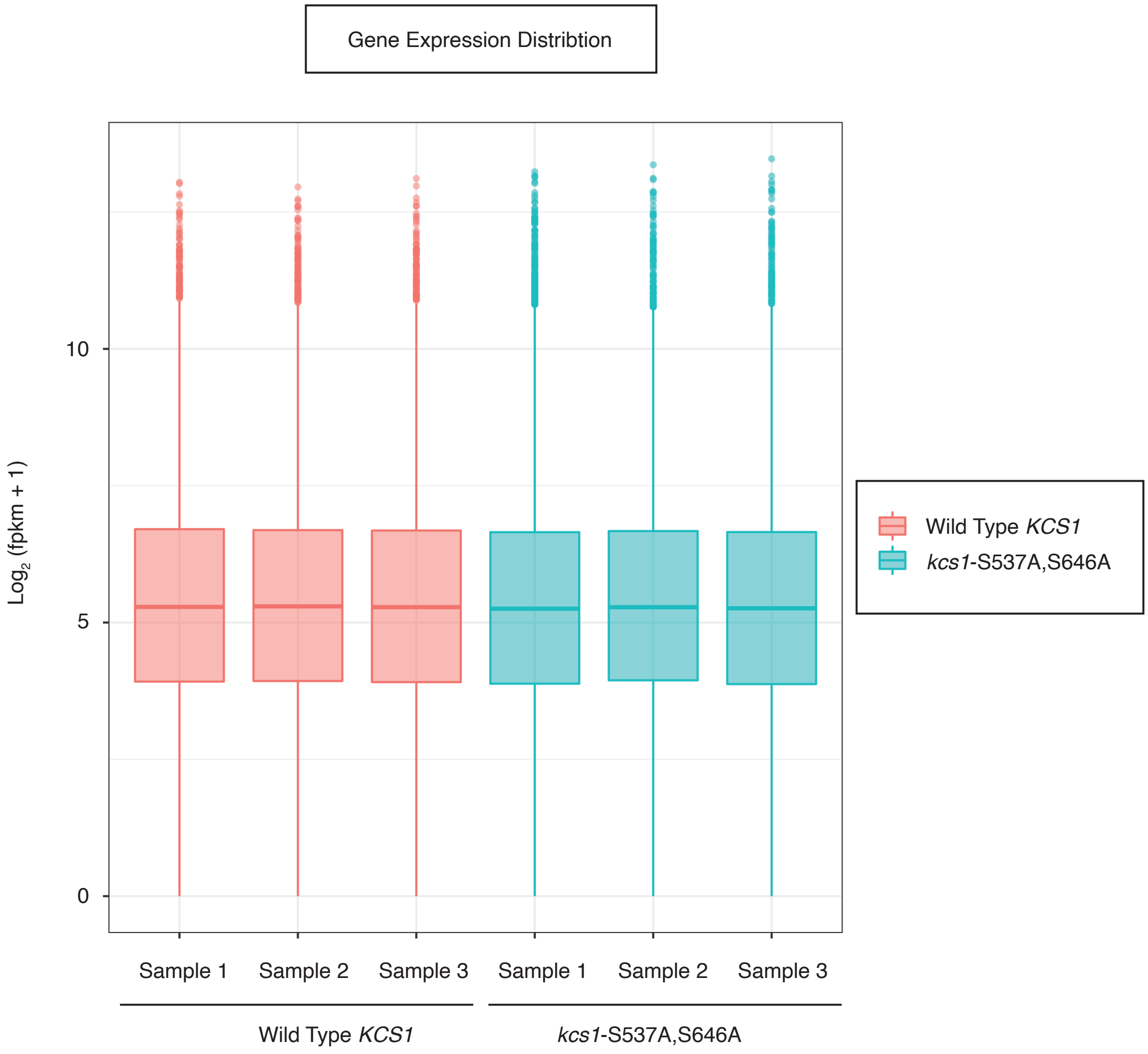

Figure S4. Gene expression distribution as determined from high-throughput sequencing of RNA samples from the control and *kcs1*-S537A,S646A strains. Boxplots are shown for the RNA sequencing results from each of three biological replicates for the two strains. The expected number of fragments per kilobase of transcript sequence per million base pairs sequenced (FPKM) was determined for each gene per sample. FPKM is calculated from the length of the given gene and the number of reads mapped to this gene. It provides an indication of the effect of sequencing depth and gene length for the reads count and is a commonly used method for the estimation of gene expression levels.

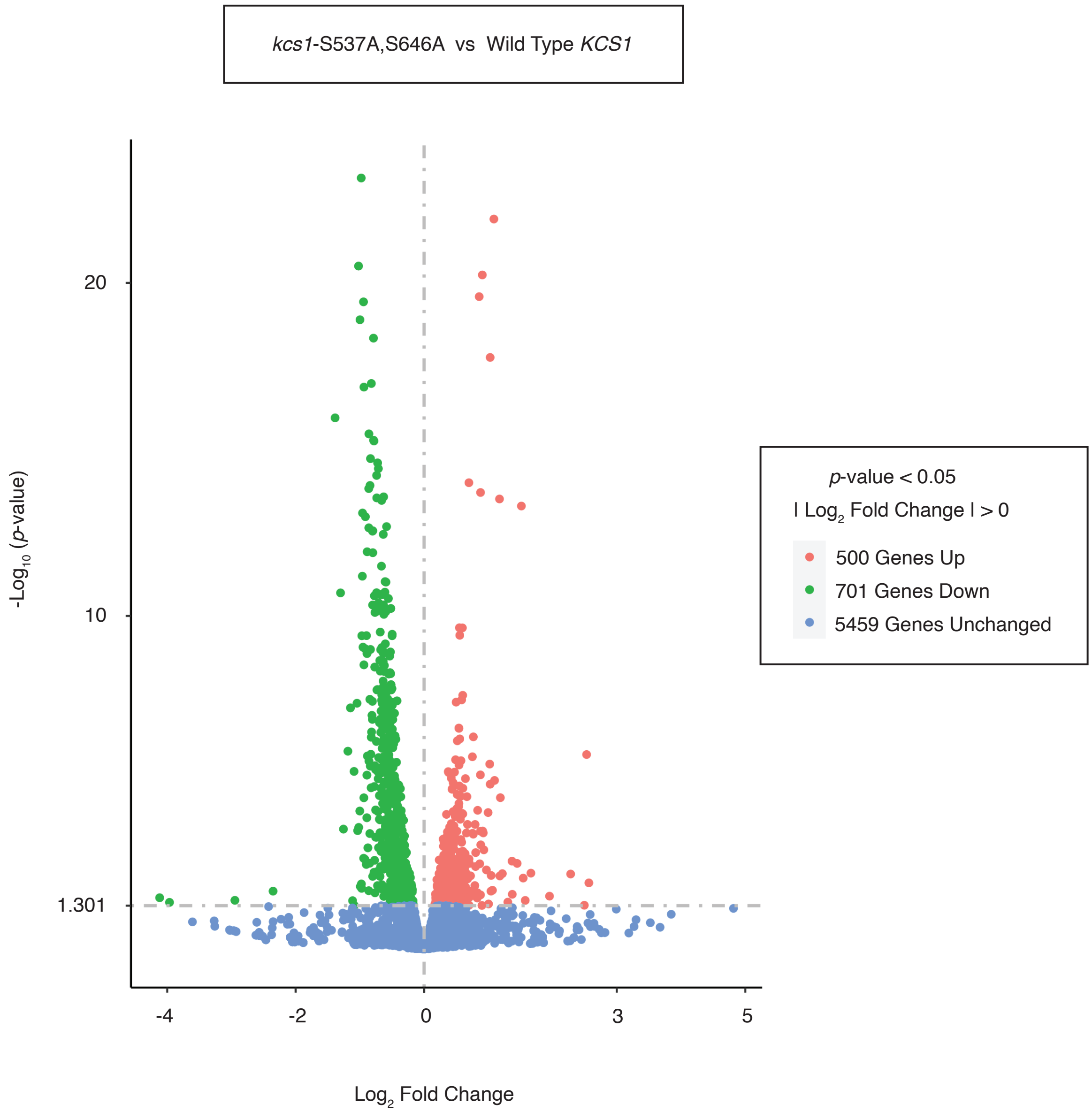

Figure S5. Volcano plot indicating the p-value versus magnitude of fold change observed for genes identified in the RNA sequencing data for the wild type and *kcs1*-S537A,S646A strains. The *p*-values are plotted on the Y-axis in $\log_{10}$ scale, and fold-change is plotted on the X-axis on a $\log_2$ scale. Values for genes are represented by dots color-coded to indicate transcript levels that are increased, decreased, or unchanged relative to levels in the strain with wild-type *KCS1*.

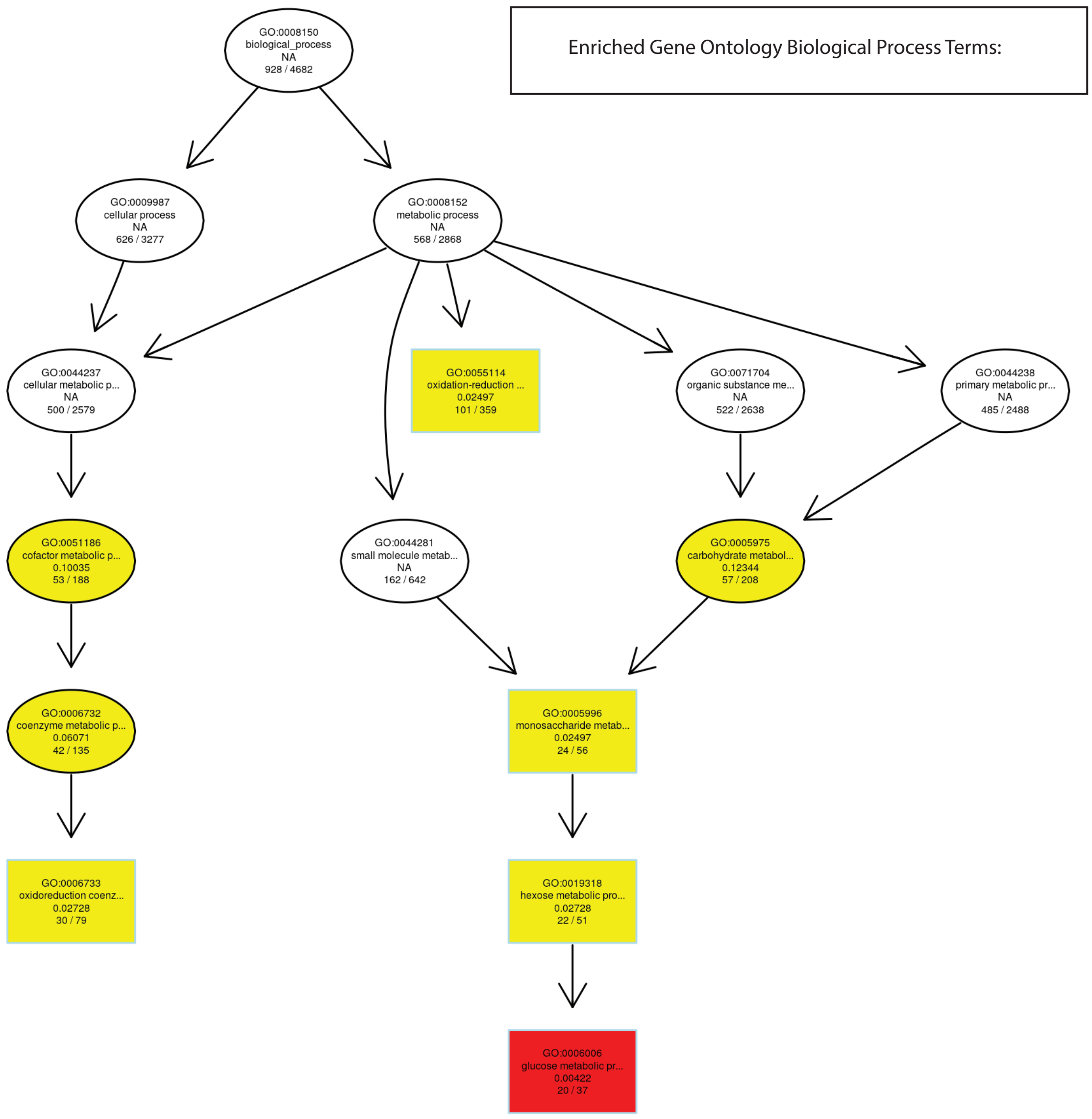

Figure S6. Tree diagram of enriched GO Biological Process terms. The diagram presents a hierarchical tree indicating Gene Ontology (GO) Biological Process terms enriched in the dataset of genes with altered transcription in *kcs1*-S537A,S646A relative to the isogenic strain with wild-type *KCS1*. Genes exhibiting either decreased or increased transcript levels in *kcs1*-S537A,S646A were included for this enrichment analysis. The GO ID and descriptor is provided in the figure, along with the *p*-value for those GO Biological Process terms that are enriched over background. The GO terms are color-coded according to the strength of the *p*-value. The number of genes with differential transcript levels annotated with the given GO term is indicated, along with the total number of genes in the yeast genome with that GO annotation. The hierarchical relationship of relevant GO Biological Process terms is illustrated by the arrows.

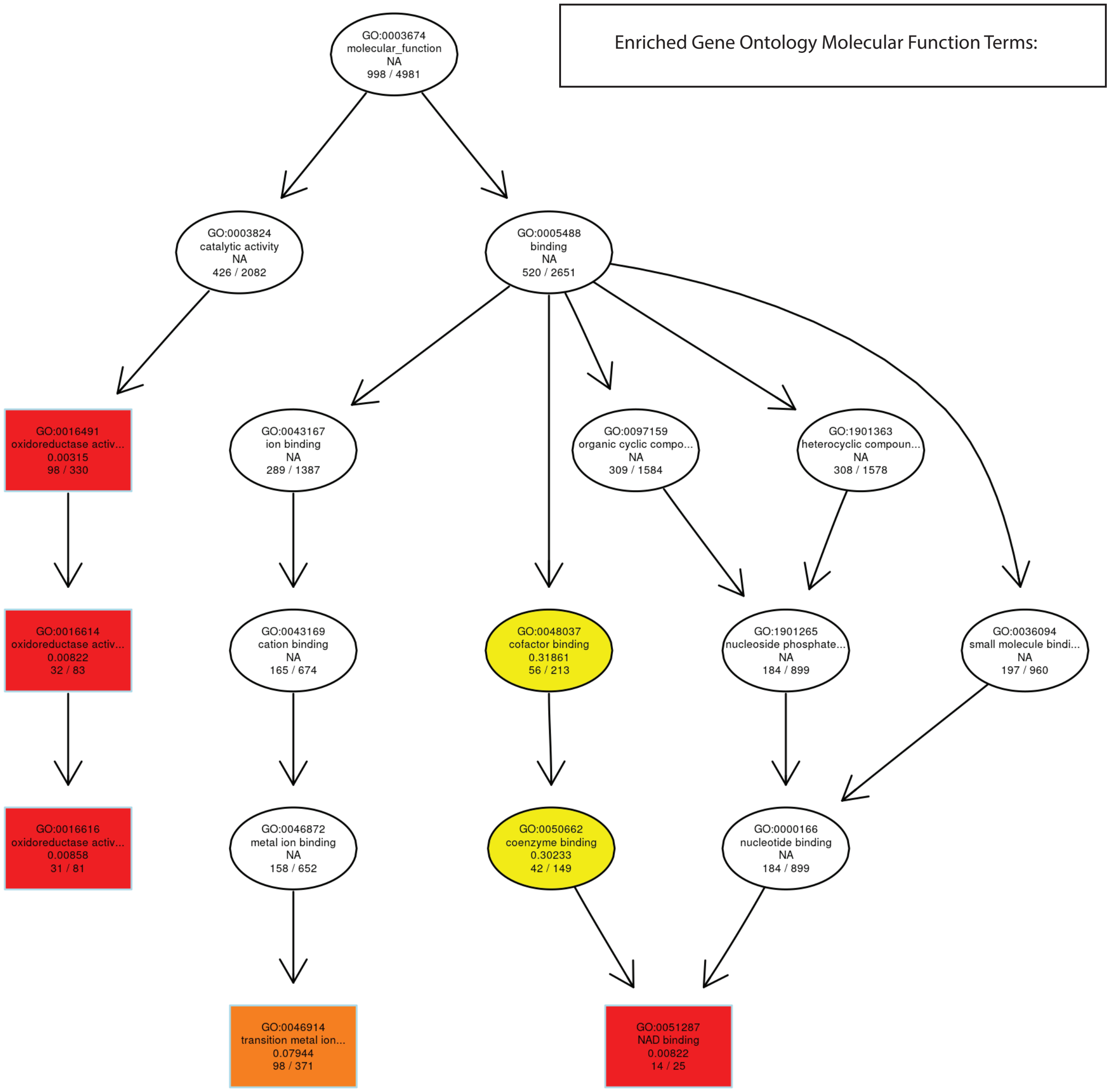

Figure S7. Diagram of enriched GO Molecular Function terms. The diagram presents a hierarchical tree indicating Gene Ontology (GO) Molecular Function terms enriched in the dataset of genes with altered transcription in *kcs1*-S537A,S646A relative to the isogenic strain with wild-type *KCS1*. This figure is presented as described in the legend to Figure S6, with the respective GO Molecular Function terms color-coded yellow to orange to red to indicate the strength of the enrichment over background (*p*-values are provided).

Table S1. List of strains used in this study.

Strain	Genotype	Source
Y825	Σ1278b *ura3-52 leu2*Δ0 *MAT*a	M. Snyder (Stanford University)
HLY337	Σ1278b *ura3-52 trp1-1 MAT*α	G. Fink (MIT)
Y825/HLY337	Σ1278b *ura3-52*/*ura3-52 leu2*Δ0 *trp1-1 MAT*a/*MAT*α	Norman *et al.* (2018)
BY4743	*his3*Δ1/*his3*Δ1 *leu2*Δ/*leu2*Δ *LYS2*/*lys2*Δ *met15*Δ/*MET15* *ura3*Δ/*ura3*Δ *MATa*/*MAT*α	M. Snyder (Stanford University)
SSB47	BL-21 + plasmids encoding *SNF1*/*SNF4*/*GAL83*/*TOS3* (2 plasmids encoding the 4 genes)	M. Schmidt (Univ. of Pittsburgh)
SSY24	Σ1278b *ura3-52 trp1-1 MAT*α + pMORF-*SNF1*	This study
SSY25	Σ1278b *ura3-52 leu2*Δ0 *MAT*a + pSGP47-*SNF1*	This study
SSY38	Σ1278b *ura3-52*/*ura3-52 leu2*Δ0 *trp1-1 MAT*a/*MAT*α *snf1*Δ::*kanMX*/snf1Δ::*kanMX*	Norman *et al.* (2018)
SSY45	Σ1278b *ura3-52*/*ura3-52 leu2*Δ0 *trp1-1 ARG82::HA-NAT*/*ARG82 MAT*a/*MAT*α	This study
SSY124	Σ1278b *ura3-52*/*ura3-52 leu2*Δ0 *trp1-1* *kcs1::HmR*/*kcs1::HmR MAT*a/*MAT*α	This study
SSY128	Σ1278b *ura3-52*/*ura3-52 leu2*Δ0 *trp1-1* *kcs1::HmR*/*kcs1::HmR MAT*a/*MAT*α + pSSB91	This study
SSY130	Σ1278b *ura3-52*/*ura3-52 leu2*Δ0 *trp1-1* *kcs1::HmR*/*kcs1::HmR MAT*a/*MAT*α + pSSB92	This study
SSY132	Σ1278b *ura3-52*/*ura3-52 leu2*Δ0 *trp1-1* *kcs1::HmR*/*kcs1::HmR MAT*a/*MAT*α + pSSB93	This study
SSY144	Σ1278b *ura3-52*/*ura3-52 leu2*Δ0 *trp1-1 kcs1*-S537A/*kcs1*-S537A *MAT*a/*MAT*α	This study
SSY146	Σ1278b *ura3-52*/*ura3-52 leu2*Δ0 *trp1-1* *kcs1::HmR*/*kcs1::HmR MAT*a/*MAT*α + pSSB96	This study

SSY148	Σ1278b *ura3-52*/*ura3-52 leu2*Δ0 *trp1-1* *kcs1::HmR*/*kcs1::HmR MAT*a/*MAT*α + pSSB95	This study

Table S2. List of plasmids used in this study.

Plasmid	Description	Source or reference
pGEX-4T-2-*SNF1*-1-392	GST-*SNF1* kinase domain AA 1-392, Amp^r	This study
pMALC2-*SNF1-KD*	Wild-type *SNF1* kinase domain, Amp^r	This study
pMALC2-*ARG82*	MBP-*ARG82* full length, Amp^r	This study
pGEX-4T-2-*MIG1*-202-414	GST-*MIG1* AA 202-414, Amp^r	This study
pMALC2-*KCS1*-1-250	MBP-*KCS1* AA 1-250, Amp^r	This study
pGEX-4T-2-*KCS1*-244-556	GST-*KCS1* AA 244-556, Amp^r	This study
pMALC2-*KCS1*-509-780	MBP-*KCS1* AA 509-780, Amp^r	This study
pMALC2-*KCS1*-755	MBP-*KCS1* AA 755-1050, Amp^r	This study
pSSB91	pRS416-*KCS1*, *URA3*, Amp^r	This study
pSSB92	pRS416-*KCS1*-S537A, *URA3*, Amp^r	This study
pSSB95	pRS416-*KCS1*-S6A, *URA3*, Amp^r	This study
pSSB96	pRS416-*KCS1*-S537A,S646A, *URA3*, Amp^r	This study
pMORF-*SNF1*	P_{gal1}-*SNF1*-His_6-HA-ZZ , 2μ, *URA3*, Amp^r	Gelperin *et al.* (2005)
pSGP47-*SNF1*	P_{adh1}-*SNF1*-His_{10}, *URA3*, Amp^r	Norman *et al.* (2018)
